# Prognosis and Medical Cost of Measuring Fractional Flow Reserve in Percutaneous Coronary Intervention

**DOI:** 10.1016/j.jacasi.2022.04.006

**Published:** 2022-07-19

**Authors:** David Hong, Seung Hun Lee, Doosup Shin, Ki Hong Choi, Hyun Kuk Kim, Taek Kyu Park, Jeong Hoon Yang, Young Bin Song, Joo-Yong Hahn, Seung-Hyuk Choi, Hyeon-Cheol Gwon, Joo Myung Lee

**Affiliations:** a Division of Cardiology, Department of Internal Medicine, Heart Vascular Stroke Institute, Samsung Medical Center, Sungkyunkwan University School of Medicine, Seoul, Republic of Korea; b Division of Cardiology, Department of Internal Medicine, Chonnam National University Hospital, Chonnam National University Medical School, Gwangju, Republic of Korea; c Division of Cardiovascular Medicine, Department of Internal Medicine, University of Iowa Carver College of Medicine, Iowa City, Iowa, USA; d Department of Internal Medicine and Cardiovascular Center, Chosun University Hospital, University of Chosun College of Medicine, Gwangju, Republic of Korea

**Keywords:** fractional flow reserve, percutaneous coronary intervention, prognosis, stable ischemic heart disease, unstable angina, CABG, coronary artery bypass graft, FFR, fractional flow reserve, IHD, ischemic heart disease, MI, myocardial infarction, PCI, percutaneous coronary intervention, PS, propensity score, RCT, randomized controlled trial

## Abstract

**Background:**

There are limited data regarding comparative prognosis and medical cost between fractional flow reserve (FFR)–based and angiography-based percutaneous coronary intervention (PCI) among revascularized patients.

**Objectives:**

This study evaluates prognosis and medical cost of FFR use in revascularized patients by PCI.

**Methods:**

Using the National Health Insurance Service database, stable or unstable angina patients who underwent PCI from 2011 to 2017 were evaluated. Eligible patients were divided into 2 groups according to use of FFR in PCI. Primary outcome was a composite of all-cause death or spontaneous myocardial infarction (MI). Secondary outcomes included individual components of the primary outcome, unplanned revascularization, and medical costs.

**Results:**

Among 134,613 eligible patients, PCI was performed based on angiography (n = 129,497) and FFR (n = 5,116). During the study period, both the annual number and proportion of use of FFR in PCI increased (all *P* for trend <0.001). The FFR group showed significantly lower risk of the primary outcome (7.0% vs 9.5%; *P* < 0.001), all-cause death (5.8% vs 7.7%; *P =* 0.001), and spontaneous MI (1.6% vs 2.2%; *P =* 0.022) than the angiography group. Although the FFR group showed higher medical cost during index admission than angiography group (median: $6,265.10 vs $5,385.60; *P* < 0.001), cumulative medical cost after index admission was significantly lower ($2,696.50 vs. $3,142.10; *P* < 0.001).

**Conclusions:**

Use of FFR in PCI in stable or unstable angina patients showed significantly lower risk of all-cause death and spontaneous MI compared to angiography-based PCI. Although the FFR group had higher initial medical cost than the angiography group, cumulative medical cost after index admission was significantly lower.

The fundamental goal of percutaneous coronary intervention (PCI) is relieving myocardial ischemia, not simply alleviating local stenosis; therefore, ischemia-directed PCI has been a standard care for patients with ischemic heart disease (IHD). Given the low diagnostic yield of noninvasive tests, fractional flow reserve (FFR) has been used as a reference surrogate marker of myocardial ischemia and FFR-guided treatment decision-making has been supported by Class Ia recommendation from guidelines.^1^^,^^2^ The DEFER (Deferral Versus Performance of Percutaneous Coronary Intervention of Functionally Nonsignificant Coronary Stenosis) trial confirmed long-term safety of deferring revascularization for stenosis with nonischemic range of FFR.^3^ The FAME (Fractional Flow Reserve Versus Angiography for Multivessel Evaluation) and FAME 2 trials have shown that PCI was beneficial only in the presence of functionally significant stenosis assessed by FFR.^4^^,^^5^ Results from a recent individual patient data meta-analysis presented the reduced risk of death or myocardial infarction with FFR-guided PCI than medical treatment alone.^6^

Nevertheless, the penetration rate of FFR in contemporary practice remains limited for multifactorial reasons such as physician attitude, knowledge barrier, and environmental barrier (ie, reimbursement, procedural time, or medical cost).^7^^,^^8^ Despite compelling evidence from randomized controlled trials (RCTs), physicians have not adopted FFR in daily practice as readily. Likely reasons for this resistance include questioning whether FFR-based PCI could reduce patient mortality, as well as not recognizing the cost savings in the long term.

To fill this gap and provide evidence to support the daily use of FFR, the current study analyzed a nationwide database and compared prognosis and medical cost between FFR-based and angiography-based PCI in patients with stable IHD or unstable angina.

## Methods

### Study design and data

This study analyzes nationwide cohort data. The data were acquired from the National Health Insurance Service (NHIS) and nationwide administrative claims database (Health Insurance Review and Assessment [HIRA] service) in the Republic of Korea.^9^^,^^10^ The NHIS is a national single-insurer in Korea which covers the entire Korean population. The NHIS and HIRA database contains information about patient demographics, diagnoses, drug prescriptions, procedures, use of medical devices including pressure wire and type of stents, medical costs, and mortality. For each patient, the clinical and claims data were longitudinally recorded. Diagnoses were coded using the International Classification of Disease-10th revision. Details about the codes used to define study population, past medical histories, medications, procedures, and devices used in PCI are summarized in Supplemental Table 1. This study was approved by the Institutional Review Board of Samsung Medical Center. Informed consent was waived as de-identified data were used.

### Study population

**Figure 1:**
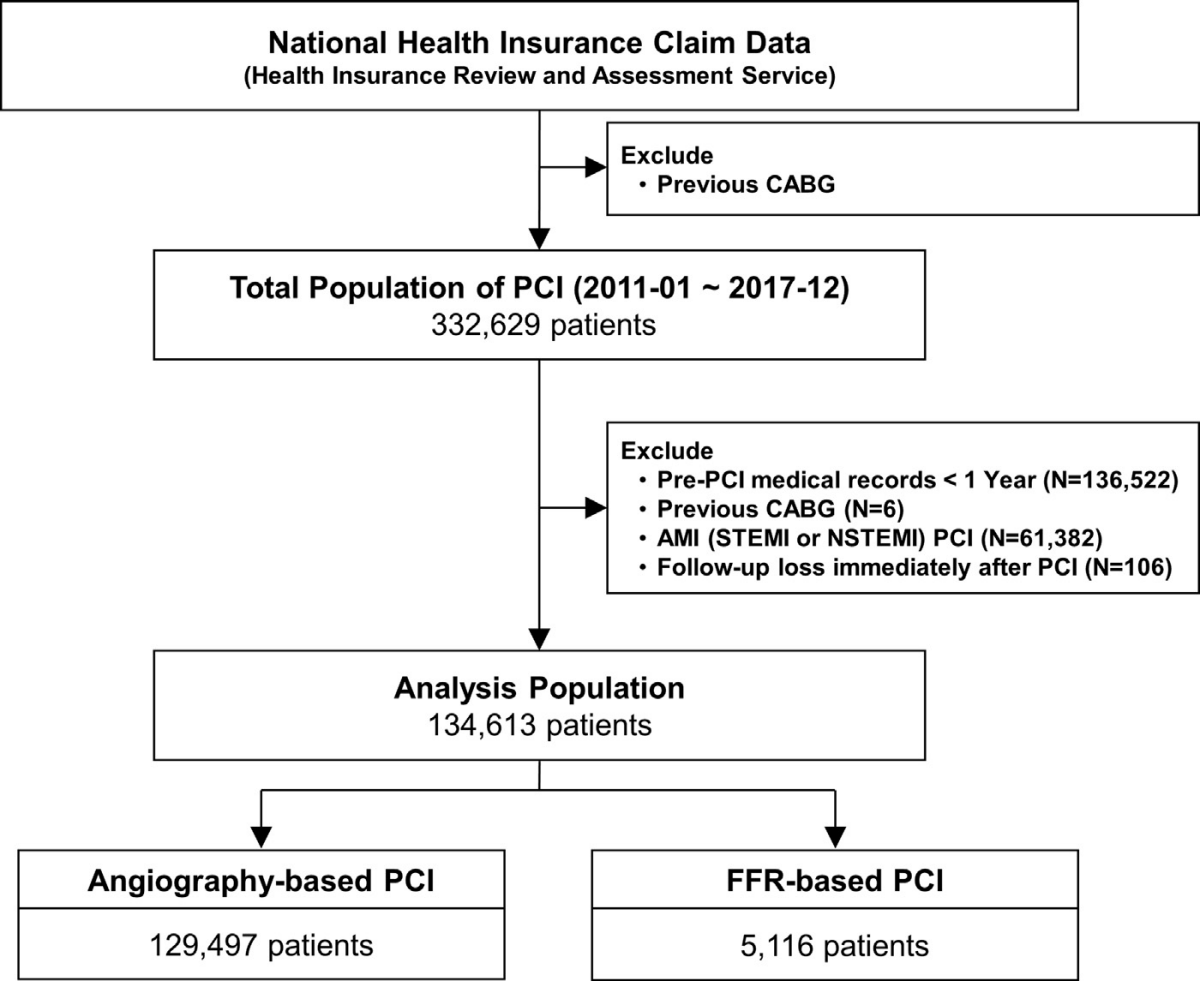
Study Flow Among 134,613 patients who underwent percutaneous coronary intervention (PCI) for stable ischemic heart disease or unstable angina from January 2011 to December 2017, PCI was performed based on angiography (n = 129,497) and fractional flow reserve (FFR) (n = 5,116).AMI = acute myocardial infarction; CABG = coronary artery bypass graft NSTEMI = non–ST-segment elevation myocardial infarction; STEMI = ST-segment elevation myocardial infarction.

We identified revascularized patients by PCI for IHD at secondary or tertiary hospitals between January 2011 and December 2017 (Figure 1). Index PCI was defined as the first PCI that was performed during this period. To investigate underlying comorbidities before the index PCI, patients who had medical records from <1 year before the index PCI were excluded from the study. Patients who had the index PCI for acute myocardial infarction (MI) including ST-segment elevation MI or non-ST-segment elevation MI, had a history of coronary artery bypass graft (CABG) surgery, or lost follow-up immediately after the index PCI were also excluded. A total of 134,613 eligible patients were divided into 2 groups according to the use of FFR during index PCI. Follow-up clinical data were assessed until December 31, 2018. Median follow-up duration was 3.0 years (IQR: 1.8 to 4.6 years).

### Clinical outcomes

The primary outcome was a composite of all-cause death or spontaneous MI during the follow-up period. Secondary outcomes included individual components of the primary outcome, unplanned revascularization, and medical costs during index admission and the follow-up period. This study uses all-cause death to minimize ascertainment bias in assessment for cause of death. Spontaneous MI was defined as receiving PCI or CABG with newly diagnosed MI after the index PCI. Unplanned revascularization was defined as receiving PCI or CABG after 1 month from the index procedure.

### Statistical analysis

All discrete or categorical variables are presented as numbers and relative frequencies (percentages), and continuous variables as mean ± SD or median with IQR (Q1 to Q3) according to distribution based on the Kolmogorov-Smirnov test and visual inspection of Q-Q plots. Linear regression analysis and Cochran-Armitage trend tests were used to analyze the time-trend of the number and proportion of FFR-based PCI, respectively. Cumulative incidence of clinical events was presented as Kaplan-Meier estimate and compared using a log-rank test. Multivariable Cox proportional hazards regression was used to calculate adjusted HR and 95% CI to compare the risk of clinical events according to the use of FFR during index PCI. Adjusted covariables were age, sex, clinical presentation, hypertension, diabetes mellitus, hyperlipidemia, congestive heart failure, previous cerebrovascular accident, atrial fibrillation, peripheral vascular disease, chronic obstructive pulmonary disease, chronic renal failure, type of stent, number of stents, discharge medications, and medical cost during index admission. The assumption of proportionality was assessed graphically by log-minus-log plot, and Cox proportional hazards models for all clinical outcomes satisfied the proportional hazards assumption. To identify independent predictor(s) of the primary outcome, the multivariable Cox proportional hazards model was used, and C-statistics and 95% CI were calculated to validate the discriminant function of the model. Cumulative medical costs and interaction between the use of FFR and follow-up duration was assessed with a linear regression model. Because of the potential for type I error caused by multiple comparisons, findings for analyses of secondary outcomes should be interpreted as exploratory. All analyses were 2-sided, and *P <* 0.05 was considered statistically significant. Statistical analyses were performed using R version 3.5.1 (R Foundation for Statistical Computing).

### Sensitivity analyses

To minimize confounding effect, sensitivity analyses were performed. First, inverse probability weighted (IPW) Cox proportional hazards regression was used to compare between group differences in clinical outcomes. Propensity-score (PS) of FFR use during index PCI was assessed by a multivariable logistic regression model. All variables, except those collected after PCI, were included in this model. For the IPW adjustment, the inverse of propensity score was adjusted in the Cox proportional hazards regression model. Second, PS-matched cohort analysis was used to compare between group differences in clinical outcomes. PS matching was performed to nearest neighbor in a 1:3 fashion without replacements. The balance of covariables between the 2 groups after PS matching was assessed by calculating standardized mean differences. Standardized mean differences of <0.1 were considered well balanced. Third, subgroup analyses were conducted by age, sex, diabetes mellitus, hyperlipidemia, atrial fibrillation, congestive heart failure, peripheral vascular disease, chronic renal failure, clinical diagnosis (stable IHD vs unstable angina), and type of used devices (drug-eluting stent vs others). Tests for interaction using multivariable Cox models were conducted to assess subgroup differences in the comparative effect of FFR use during index PCI.

## Results

### Patients and procedural characteristics

**Figure 2:**
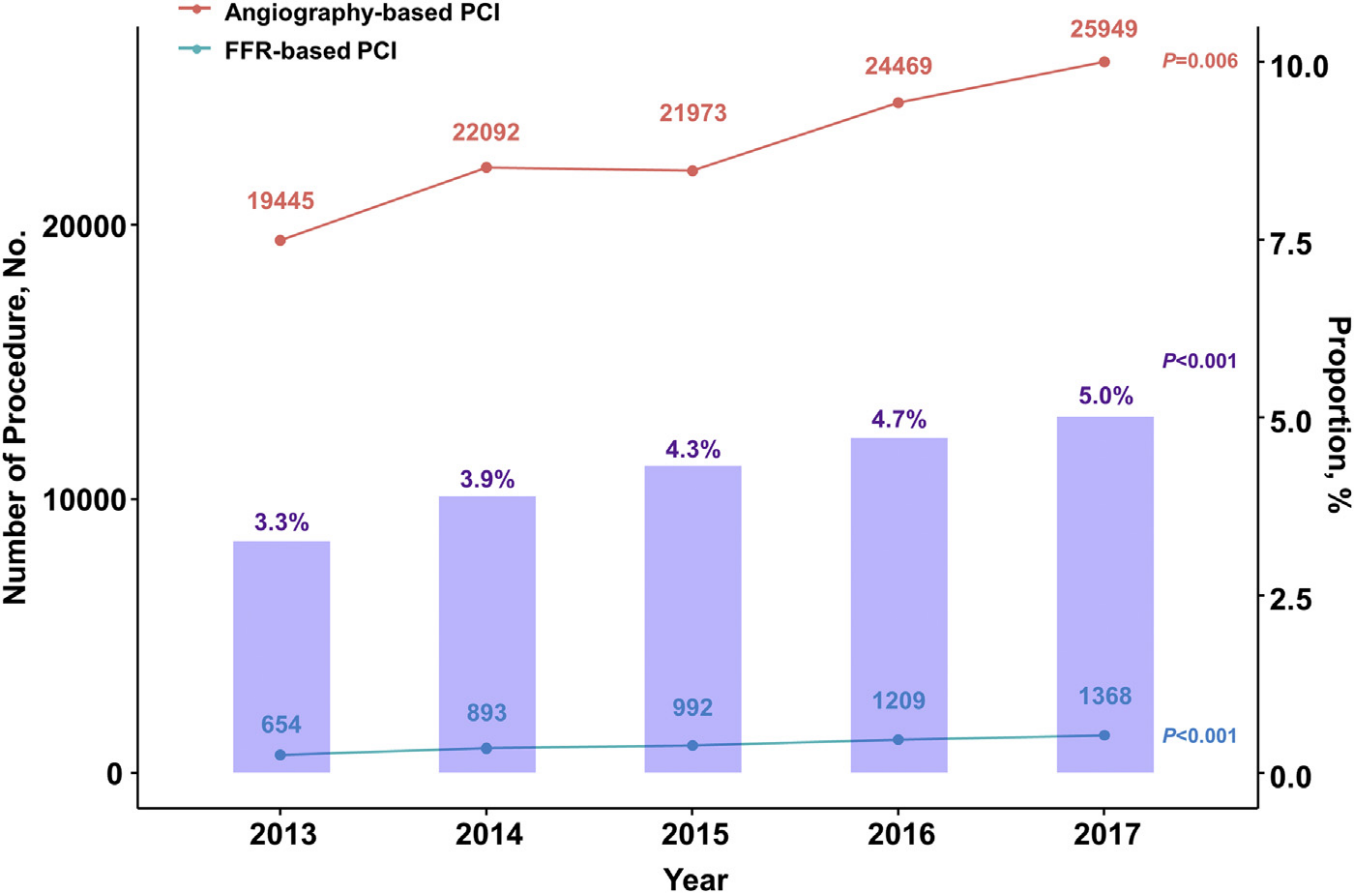
Annual Trends of Adoption Rates of FFR-Based PCI Both annual number and proportion of FFR-based PCI significantly increased during the study period. **Red dots and red line** indicate annual number of angiography-based PCI. **Blue dots and blue line** indicate annual number of FFR-based PCI. **Purple boxes** represent proportion of FFR-based PCI in total number of PCI. Abbreviations as in Figure 1.

Among 134,613 eligible patients who underwent PCI for stable IHD or unstable angina, PCI was performed based on angiographic findings in 129,497 patients (96.2%). Conversely, FFR was used during index PCI in 5,116 patients (3.8%) (Figure 1). Patient and procedural characteristics are presented in Table 1. At index PCI, 51.6% and 48.4% of patients presented with stable and unstable angina, respectively. The majority of PCIs (90.7%) were performed using second-generation drug-eluting stents. The study population was treated by guideline-directed medical treatment before and after the index PCI. When patient characteristics between the 2 groups were compared, the FFR group was younger, and more likely to be male, and to present with stable angina than the angiography group. Conversely, the FFR group had higher prevalence of hypertension, diabetes, and hyperlipidemia than the angiography group. Regarding procedural characteristics, the number of stents used was slightly higher in the FFR group than in the angiography group (Table 1). Both the annual number (654 per year to 1,368 per year, *P* for trend <0.001) and proportion (3.3% to 5.0%, *P* for trend <0.001) of FFR-based PCI significantly increased during the study period (Figure 2). Although the number of angiography-based PCI has increased (19,445 per year to 25,949 per year, *P* for trend = 0.006), the proportion of angiography-based PCI among total volume of PCI has gradually decreased (96.8% to 94.9%, *P* for trend < 0.001).

**Table 1:** Patient and Procedural Characteristics of Study Population

	Total (N = 134,613)	Angiography-Based PCI (n = 129,497)	FFR-Based PCI (n = 5,116)	*P* Value
Demographics				
Mean age, y	66.8 ± 10.3	66.9 ± 10.3	65.7 ± 10.0)	<0.001
Female	45,912 ± 34.1	44,353 ± 34.2	1,559 ± 30.5)	<0.001
Clinical diagnosis				<0.001
Stable ischemic heart disease	69,424 (51.6)	66,195 (51.1)	3,229 (63.1)	
Unstable angina	65,189 (48.4)	63,302 (48.9)	1,887 (36.9)	
Cardiovascular risk factors				
Hypertension	96,480 (71.7)	92,735 (71.6)	3,745 (73.2)	0.014
Diabetes mellitus	66,309 (49.3)	63,666 (49.2)	2,643 (51.7)	<0.001
Hyperlipidemia	93,707 (69.6)	89,863 (69.4)	3,844 (75.1)	<0.001
Atrial fibrillation	9,290 (6.9)	8,975 (6.9)	315 (6.2)	0.035
Congestive heart failure	29,697 (22.1)	28,548 (22.0)	1,149 (22.5)	0.495
Chronic renal failure	12,228 (9.1)	11,812 (9.1)	416 (8.1)	0.017
Previous CVA	37,509 (27.9)	36,115 (27.9)	1,394 (27.2)	0.324
Peripheral vascular disease	26,652 (19.8)	25,635 (19.8)	1,017 (19.9)	0.898
Chronic obstructive pulmonary disease	21,945 (16.3)	21,122 (16.3)	823 (16.1)	0.685
Liver disease	32,583 (26.2)	33,922 (29.5)	1,361 (26.6)	0.526
Dementia	1,522 (1.1)	1,472 (1.1)	50 (1.0)	0.322
Baseline medications				
Aspirin	75,910 (56.4)	72,749 (56.2)	3,161 (61.8)	<0.001
P2Y_12_ inhibitor	55,980 (41.6)	53,799 (41.5)	2,181 (42.6)	0.126
Anticoagulant (warfarin or NOAC)	4,178 (3.1)	4,039 (3.1)	139 (2.7)	0.113
ACEI or ARBs	54,797 (40.7)	52,743 (40.7)	2,054 (40.1)	0.415
Beta blocker	47,845 (35.5)	45,860 (35.4)	1,985 (38.8)	<0.001
Calcium channel blocker	52,252 (38.8)	50,153 (38.7)	2,099 (41.0)	0.001
Nitrate	34,368 (25.5)	32,976 (25.5)	1,392 (27.2)	0.005
Statin	80,800 (52.6)	67,758 (52.3)	3,042 (59.5)	<0.001
Ezetimibe	9,088 (6.8)	8,566 (6.6)	522 (10.2)	<0.001
Fenofibrate	5,257 (3.9)	5,029 (3.9)	228 (4.5)	0.041
Medications after index procedure				
Aspirin	123,092 (91.4)	118,286 (91.3)	4,806 (93.9)	<0.001
P2Y_12_ inhibitor	124,521 (92.5)	119,650 (92.4)	4,871 (95.2)	<0.001
Clopidogrel	120,104 (89.2)	115,402 (89.1)	4,702 (91.9)	<0.001
Ticagrelor or Prasugrel	15,079 (11.2)	14,480 (11.2)	599 (11.7)	0.251
Anticoagulant (warfarin or NOAC)	8,364 (6.2)	8,079 (6.2)	285 (5.6)	0.056
ACEI or ARBs	87,221 (64.8)	84,094 (64.9)	3,127 (61.1)	<0.001
Beta blocker	81,809 (60.8)	78,816 (60.9)	2,993 (58.5)	0.001
Calcium channel blocker	69,738 (51.8)	67,001 (51.7)	2,737 (53.5)	0.014
Nitrate	61,116 (45.4)	59,029 (45.6)	2,087 (40.8)	<0.001
Statin	122,032 (90.7)	117,238 (90.5)	4,794 (93.7)	<0.001
Ezetimibe	26,856 (20.0)	25,527 (19.7)	1,329 (26.0)	<0.001
Fenofibrate	5,325 (4.0)	5,156 (4.0)	169 (3.3)	0.016
Procedure characteristics				
Number of stent used	1.46 ± 0.76	1.46 ± 0.76	1.50 ± 0.79	0.001
Type of device used				<0.001
Drug-eluting stent	122,152 (90.7)	117,424 (90.7)	4,728 (92.4)	
Drug-coated balloon angioplasty	3,344 (2.5)	3,240 (2.5)	104 (2.0)	
Plain old balloon angioplasty	7,250 (5.4)	7,085 (5.5)	165 (3.2)	
Bare metal stent	874 (0.6)	852 (0.7)	22 (0.4)	
Bioresorbable vascular scaffold	993 (0.7)	896 (0.7)	97 (1.9)	
Medical cost, $^a^				
Index admission, $				
Median cost	5,442.1 (4,601.7-7,218.8)	5,385.6 (4,586.5-7,181.4)	6,265.1 (5,440.1-7,967.6)	<0.001
Mean cost	6,335.7 ± 3,135.6	6,304.2 ± 3,135.9	7,134.1 ± 3,019.9	<0.001
During follow-up period, $				
Median cost	3,125.1 (1,178.4-8,021.0)	3,142.1 (1,183.9-8,070.0)	2,696.5 (1,049.8-6,963.3)	<0.001
Mean cost	9,297.6 ± 18,184.5	9,362.7 ± 18,242.6	7,649.4 ± 16,559.9	<0.001

Values are mean ± SD, n (%), or median (IQR).

ACEI = angiotensin-converting enzyme inhibitor; ARB = angiotensin receptor blocker; CVA = cerebrovascular accident; FFR = fractional flow reserve; NOAC = non–vitamin K antagonist oral anticoagulant; PCI = percutaneous coronary intervention.

a Medical cost in Korean Won was converted to US Dollars (1126.56 Korean Won, ₩ = 1 dollar, $).

### Comparative prognosis between FFR-based and angiography-based PCI

**Figure 3:**
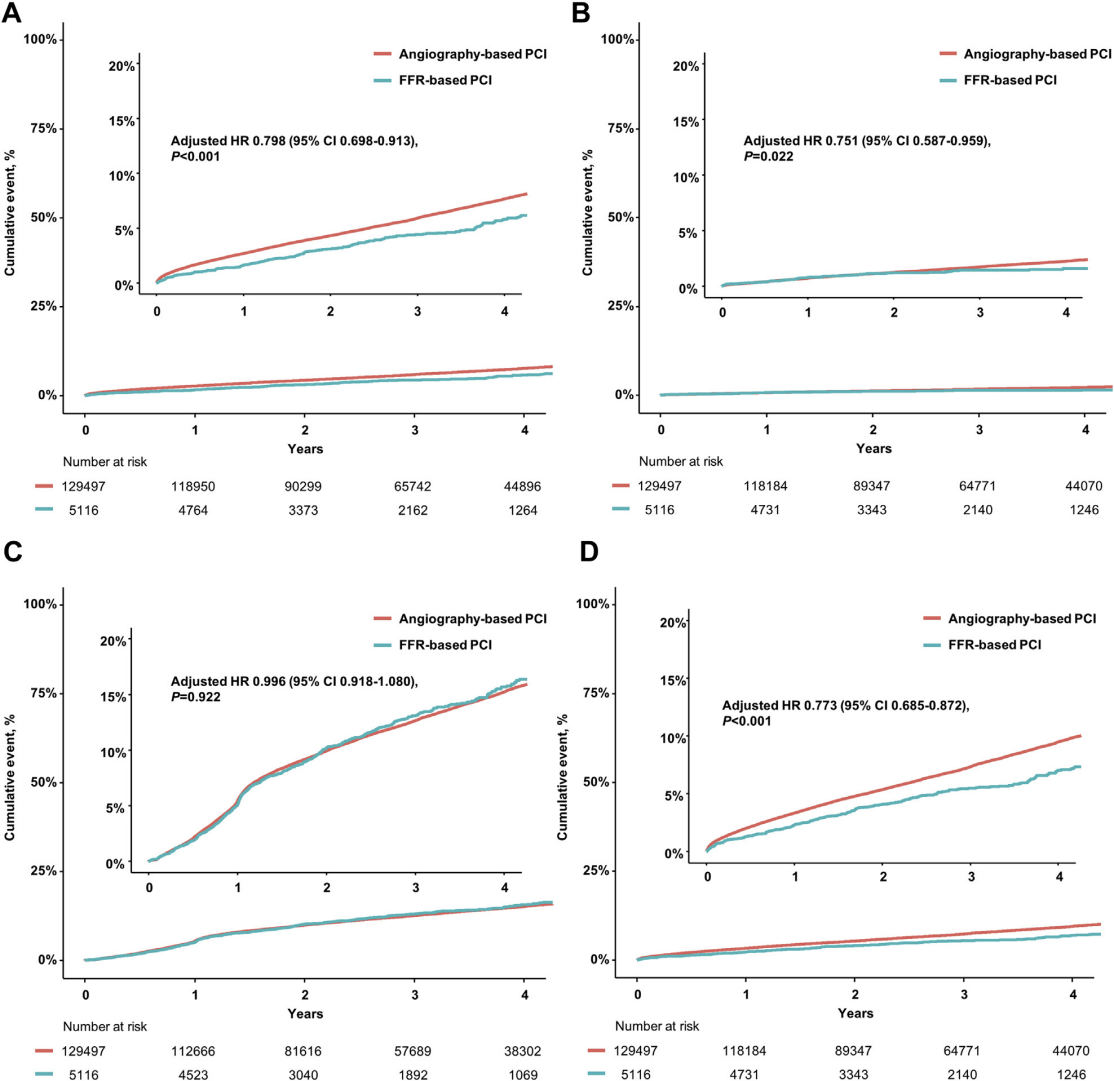
Cumulative Incidence of Clinical Events Between Angiography and FFR-Based PCI Cumulative incidence of **(A)** all-cause death, **(B)** spontaneous myocardial infarction, **(C)** unplanned revascularization, and **(D)** death or spontaneous myocardial infarction were compared between FFR-based PCI and angiography-based PCI. Abbreviations as in Figure 1.

During the follow-up period, a total of 7,737 deaths and 2,179 spontaneous MIs occurred (Table 2, Figure 3). The FFR group showed significantly lower risk of the primary outcome (7.0% vs 9.5%; adjusted HR: 0.773; 95% CI: 0.685-0.872; *P <* 0.001) than the angiography group, which was driven by the lower risk of both all-cause death (5.8% vs 7.7%; adjusted HR: 0.798; 95% CI: 0.698-0.913; *P =* 0.001), and spontaneous MI (1.6% vs 2.2%; adjusted HR: 0.751; 95% CI: 0.587-0.959; *P =* 0.022) in the FFR group than in the angiography group. There was no significant difference in the risk of unplanned revascularization between the 2 groups (15.7% vs 15.2%; adjusted HR: 0.996; 95% CI: 0.918-1.080; *P =* 0.922).

**Table 2:** Clinical Outcomes Between Angiography-Based and FFR-Based PCI

	Total (N = 134,613)	Angio-Based PCI (n = 129,497)	FFR-Based PCI (n = 5,116)				
				Unadjusted	Multivariable^a^	IPW-Adjusted	PS-Matched
All-cause death	7,737 (7.6)	7,532 (7.7)	205 (5.8)	0.724 (0.633-0.828); *P* < 0.001	0.798 (0.698-0.913); *P* = 0.001	0.714 (0.624-0.817); *P* < 0.001	0.716 (0.618-0.829); *P* < 0.001
Spontaneous MI	2,179 (2.2)	2,115 (2.2%)	64 (1.6)	0.778 (0.609-0.993); *P* = 0.044	0.751 (0.587-0.959); *P* = 0.022	0.776 (0.608-0.990); *P* = 0.041	0.756 (0.578-0.990); *P* = 0.042
Unplanned revascularization	15,733 (15.2)	15,147 (15.2)	586 (15.7)	1.013 (0.934-1.098); *P* = 0.752	0.996 (0.918-1.080); *P* = 0.922	1.015 (0.937-1.101); *P* = 0.711	1.040 (0.949-1.139); *P* = 0.406
Death or spontaneous MI	9,598 (9.4)	9,343(9.5)	255 (7.0)	0.722 (0.640-0.815); *P* < 0.001	0.773 (0.685-0.872); *P* < 0.001	0.714 (0.633-0.806); *P* < 0.001	0.717 (0.628-0.818); *P* < 0.001

Values are n (%) or HR (95% CI).

IPW = inverse probability weighting; MI = myocardial infarction; PS = propensity score; other abbreviations as in Table 1.

a Multivariable analysis included age, sex, clinical presentation, hypertension, diabetes mellitus, hyperlipidemia, congestive heart failure, previous CVA, atrial fibrillation, peripheral vascular disease, chronic obstructive pulmonary disease, chronic renal failure, type of stent, number of stents, discharge medications, and medical cost during index admission.

### Sensitivity analyses and independent predictors of all-cause death or spontaneous MI

**Figure 4:**
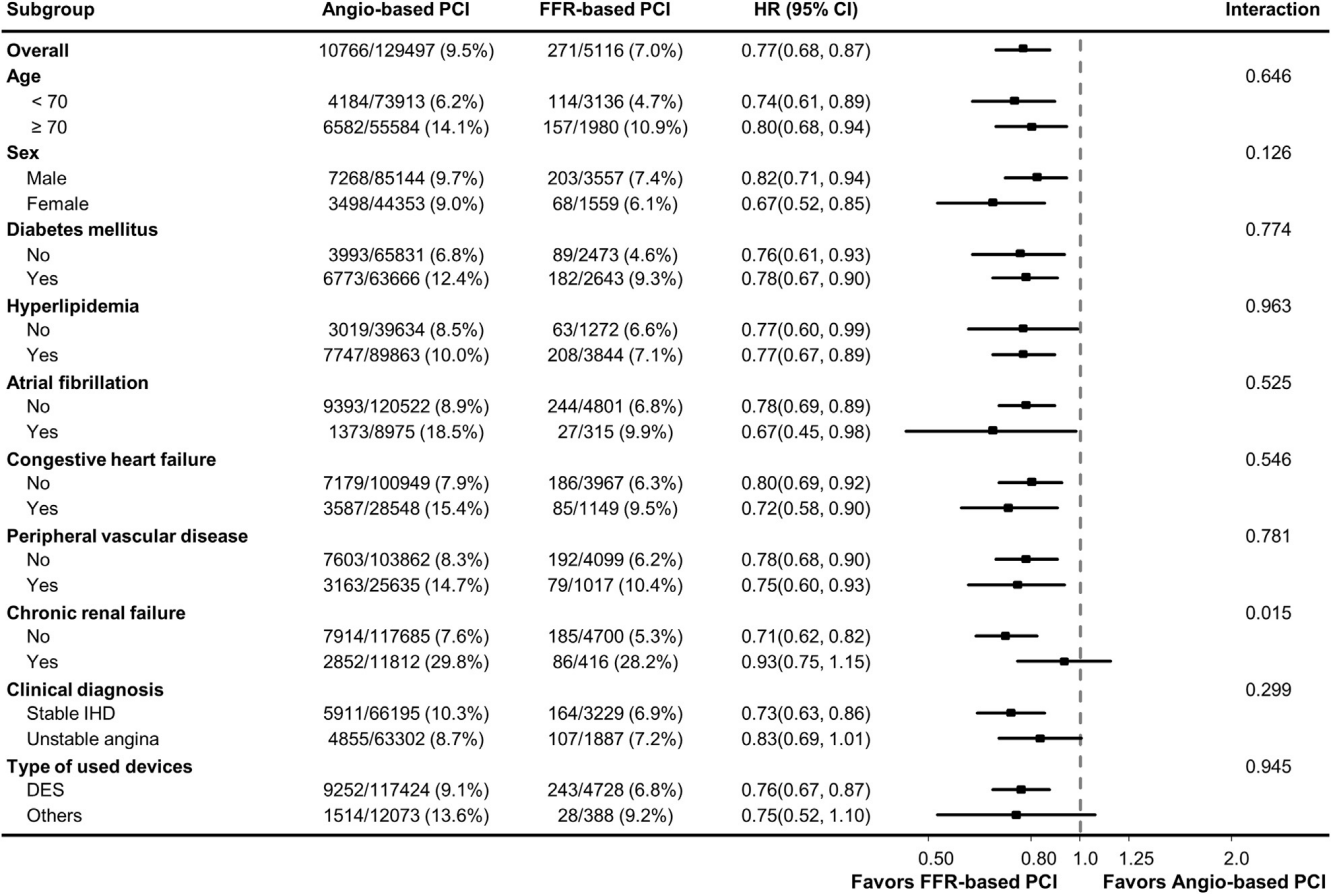
Subgroup Analysis for Death or Spontaneous Myocardial Infarction The significantly lower risk of death or spontaneous myocardial infarction in the FFR group than the angiography group was consistently observed across various subgroups. DES = drug-eluting stent; other abbreviations as in Figure 1.

In sensitivity analysis, PS matching yielded 5,116 patients in the FFR group and 15,348 patients in the angiography group. Distribution of covariables after PS matching was well balanced with standardized mean differences of <0.1 (Supplemental Table 2). In comparison of clinical outcomes between the FFR and angiography groups, IPW adjustment and PS-matched group analysis showed that the risk of the primary outcome, all-cause death, and spontaneous MI were significantly lower in the FFR group than in the angiography group (Table 2). The significantly lower risk of the primary outcome in the FFR group than in the angiography group was consistently observed across various subgroups without significant interaction *P* values, except in patients with chronic renal failure (Figure 4). In addition, FFR use at index PCI was independently associated with a decreased risk of the primary outcome (HR: 0.850; 95% CI: 0.750-0.963; *P =* 0.010) (Table 3).

**Table 3:** Independent Predictors for All-Cause Death or Spontaneous MI

	Univariable Analysis		Multivariable Analysis^a^	
	HR (95% CI)	*P* Value	HR (95% CI)	*P* Value
FFR use at index PCI	0.722 (0.640-0.815)	<0.001	0.850 (0.750-0.963)	0.010
Unstable angina	0.836 (0.803-0.871)	<0.001	0.991 (0.952-1.032)	0.668
Age, y	1.057 (1.054-1.059)	<0.001	1.055 (1.052-1.057)	<0.001
Female	0.927 (0.888-0.967)	<0.001	0.820 (0.785-0.857)	<0.001
Hypertension	1.799 (1.708-1.895)	<0.001	1.086 (1.024-1.152)	0.006
Diabetes mellitus	1.884 (1.808-1.964)	<0.001	1.293 (1.235-1.354)	<0.001
Hyperlipidemia	1.173 (1.122-1.227)	<0.001	0.867 (0.825-0.911)	<0.001
Atrial fibrillation	2.225 (2.097-2.362)	<0.001	1.509 (1.420-1.603)	<0.001
Chronic renal failure	4.485 (4.288-4.691)	<0.001	2.520 (2.397-2.649)	<0.001
Number of stents used	1.112 (1.084-1.140)	<0.001	1.152 (1.122-1.182)	<0.001
Drug-eluting stent	0.640 (0.603-0.678)	<0.001	0.691 (0.651-0.734)	<0.001
Medications after index procedure				
Aspirin	0.187 (0.179-0.195)	<0.001	0.712 (0.652-0.778)	<0.001
P2Y_12_ inhibitor	0.173 (0.165-0.181)	<0.001	0.660 (0.602-0.723)	<0.001
ACEI or ARBs	0.537 (0.517-0.559)	<0.001	0.855 (0.814-0.899)	<0.001
Beta blocker	0.658 (0.632-0.685)	<0.001	1.089 (1.037-1.143)	<0.001
Statin	0.175 (0.167-0.182)	<0.001	0.461 (0.427-0.497)	<0.001

Abbreviations as in Tables 1 and 2.

a Discriminant ability of the multivariable model was 0.762 (95% CI: 0.756-0.768).

### Comparative medical cost between FFR-based and angiography-based PCI

**Figure 5:**
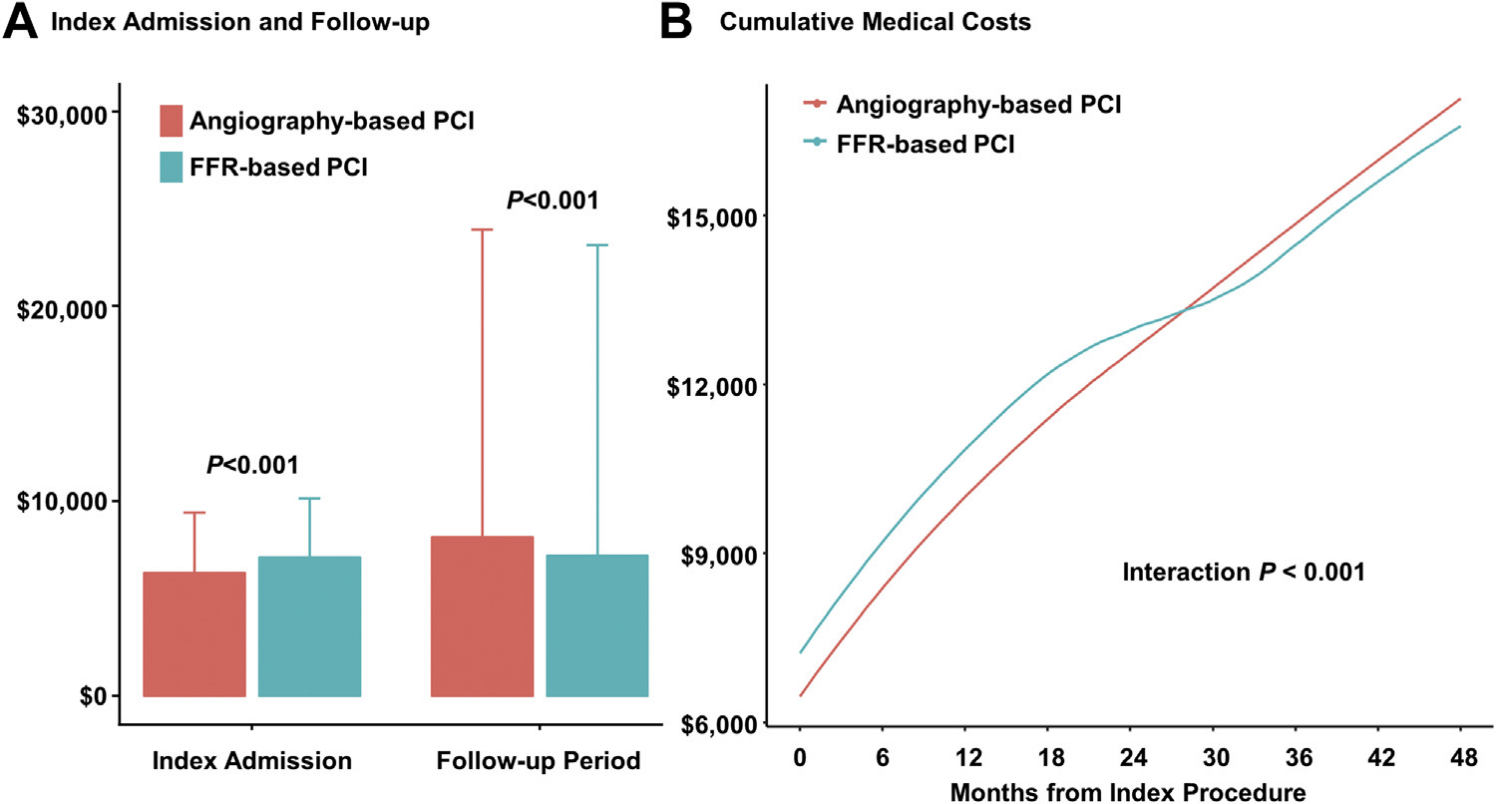
Comparison of Cumulative Medical Cost between Angiography and FFR-Based PCI **(A)** Medical costs during index admission and follow-up after the index admission are compared between FFR-based PCI and angiography-based PCI. **(B)** There was significant interaction between FFR use during index PCI and follow-up duration (interaction *P <* 0.001). Mean values of medical costs were plotted and compared between angiography and FFR-based PCI. Abbreviations as in Figure 1.

Although the FFR group showed higher medical cost during the index admission than the angiography group (median: $6,265.1 vs $5,385.6; mean: $7,134.1 vs $6,304.2; *P <* 0.001). The curves of cumulative medical cost had crossed at approximately 24 to 30 months after the index procedure. Total cumulative medical cost during 4-year follow-up period after the index admission was significantly lower in the FFR group than in the angiography group (median: $2,696.5 vs $3,142.1; mean: $7,649.4 vs $9,362.7; *P <* 0.001) (Table 1, Figure 5). These changes in cumulative medical costs during the follow-up period were significantly associated with the use of FFR during PCI (interaction *P* < 0.001) (Figure 5).

## Discussion

The current study evaluated a nationwide cohort to compare prognosis and cumulative medical cost according to the use of FFR during index PCI. The main findings were as follows: First, both the annual number and proportion of FFR-based PCI have significantly increased. Second, FFR-based PCI was significantly associated with lower risk of all-cause death or spontaneous MI than angiography-based PCI. However, there was no significant difference in the risk of unplanned revascularization between the 2 groups. The results were consistent in various sensitivity analyses. Third, although the FFR group showed higher medical cost during index admission than the angiography group, cumulative medical cost during the follow-up period after the index admission was significantly lower in the FFR group than in the angiography group, mainly caused by lower risk of adverse clinical events.

### Comparative prognosis according to the use of FFR at index PCI

The presence and extent of myocardial ischemia are fundamental parameters for determining the need for PCI.^11^ FFR is a surrogate marker of myocardial ischemia, and previous RCTs have shown that FFR-guided PCI improves clinical outcomes in patients with IHD.^3^, ^4^, ^5^^,^^12^ However, these RCTs were underpowered to evaluate the survival benefit of FFR-guided PCI. Recently, 2 large observational studies evaluated the association between FFR-guided PCI and mortality of patients.^13^^,^^14^ Using the Swedish Coronary Angiography and Angioplasty Registry (SCAAR) registry (n = 23,680), Volz et al^14^ reported that the use of FFR was associated with a lower risk of mortality, stent thrombosis, and restenosis at median follow-up of 4.7 years. Also, Parikh et al^13^ presented the pattern of FFR use and the significantly lower risk of mortality at 1 year following FFR-guided PCI than angiography-guided PCI from the Veterans Affairs (VA) registry (n = 17,989).

Nevertheless, neither study was able to show specific outcomes or mechanisms that could support the mortality reduction. MI, a key outcome that could make a difference in mortality, was not investigated in the SCAAR registry and did not differ according to the use of FFR in the VA registry (0.64% vs 0.79% for FFR and angiography-PCI, respectively; *P =* 0.31).^13^, ^14^, ^15^ Possible reasons might be limited sample size, number of events, and a relatively short follow-up period in the VA registry. In addition, only 6.5% (1,168 of 17,989) of patients received revascularization by PCI or CABG in the VA registry. Therefore, these results are not representative of the contemporary PCI population, and overall ischemia burden of study population might be relatively low, which might explain the relatively low incidence of MI without between-group difference.

Complementing the results from previous RCTs, this nationwide cohort study showed findings consistent with those of 2 recent observational studies. This study captured all consecutive patients who underwent PCI during the study period in Korea and was the largest-scale study among studies that explored the prognostic implications of PCI based on FFR compared with PCI based on angiography. The current study showed that FFR-based PCI was significantly associated with a lower risk of mortality. In addition, significantly a lower risk of spontaneous MI could support the lower mortality in the FFR group.

### Medical cost according to the use of FFR at index PCI

The additional cost of measuring FFR is one of the main reasons for underuse of FFR. Studies on the long-term cost-effectiveness of FFR-guided PCI are limited to date. In the 5-year report of the FAME trial, FFR-guided PCI showed similar long-term prognosis with using a significantly lower number of stents during index PCI compared to angiography-guided PCI.^4^ In the FAME 2 substudy, FFR-guided PCI showed higher cost at index admission than optimal medical therapy ($9,944 vs $4,440; *P <* 0.001); however, the cumulative medical cost was similar between the 2 groups at 3 years ($16,792 vs $16,737; *P =* 0.94).^16^ Our findings are in line with previous studies. As NHIS is the sole-insurer that covers the entire national population, this study was able to thoroughly calculate all medical expenses using NHIS claim data. In this study, only patients who underwent PCI were included. As a result, compared to angiography-based PCI, FFR-based PCI had higher cost at index admission caused by the cost of pressure wire and hyperemic agents. However, in long-term follow-up, cumulative medical cost was significantly lower in the FFR group than in the angiography group. This might be caused by significantly lower risk of adverse clinical events with following medical cost. Considering that the number of stents used was higher in the FFR group, the lower risk of adverse clinical events and cumulative medical cost during follow-up period imply that FFR-based PCI could provide better lesion selection than angiography-based PCI. The current study did not include patients with deferred revascularization based on insignificant FFR because only patients who underwent PCI were evaluated. Therefore, the current study did not reflect the cost-reducing effect of FFR by preventing unnecessary PCI based on insignificant FFR. If this effect were additionally evaluated, the cost-effectiveness of FFR-guided PCI would have been even greater.

### Adoption rates and temporal trends of FFR

FFR is recommended to assess hemodynamic significance and guide PCI.^1^^,^^17^ However, the adoption rate of FFR-guided revascularization in clinical practice remains low. In most countries, adoption rates are within 6%.^7^ Similar to that of many other countries worldwide, the adoption rate of FFR in Korea was 3.8%. Although the use of FFR has been steadily increasing, it is still underused. Lack of evidence for the survival benefit and additional medical cost of FFR measurement were probably key contributors of underuse. Considering the survival benefit as well as cost-effectiveness of FFR shown in the current and previous studies, efforts are needed to increase the adoption rate of FFR to improve the long-term prognosis of patients.^4^^,^^13^^,^^14^^,^^16^ Still, obstacles such as hyperemia-related complications and prolongation of procedural time in FFR remain. Nonhyperemic pressure ratios such as instantaneous wave-free ratio proved noninferiority to FFR and its long-term prognostic implications.^18^, ^19^, ^20^ Furthermore, angiography-derived physiologic indexes that do not require a pressure wire and hyperemic agents showed excellent diagnostic accuracy in identifying hemodynamically significant coronary stenosis.^21^^,^^22^ These convenient physiologic assessment tools will contribute to enhancing the implementation of ischemia-targeted revascularization and improving patient prognosis in IHD.

### Study limitations

First, this study was an observational study that was inherently susceptible to confounding or selection bias. However, the current study used nationwide cohort data and evaluated all consecutive patients who underwent PCI during the study period, thus, selection bias might be minimal. In addition, multiple sensitivity analyses showed consistent results. Nevertheless, unmeasured confounding bias such as operator experience and preference should be considered in interpreting these results. Second, study population, baseline characteristics, clinical presentation, and clinical outcomes were adjudicated based on administrative claim database, which can be susceptible to coding error. Nonetheless, as the NHIS routinely audits the claims, such data are considered reliable and validated in multiple peer-reviewed publications.^10^^,^^23^^,^^24^ Third, detailed lesions and procedural characteristics could not be investigated as we used an administrative claim database. To compensate for this limitation, baseline characteristics including cardiovascular risk factors and medications, which may influence lesion and procedural characteristics, were thoroughly investigated and adjusted. Fourth, patients with deferred revascularization based on FFR or angiographic findings were not included in this study. Fifth, as the proportion of FFR-based PCI significantly increased over time, the possible confounding effects from advanced medical treatment and quality of procedure, especially among large-volume hospitals with early adoption of FFR-based PCI, could partially affect the overall results. Sixth, current results were generated under national health policy of the Korean government, especially for medical cost, because the NHIS controls all medical costs, from devices to doctors’ fees. Therefore, caution is needed when extrapolating the current results to another country with different health care policies. Additionally, although NHIS and HIRA data included the details of claimed medical cost, it could not be differentiated the medical costs as pre- or post-procedural stage. Cumulative medical costs after index procedure included both costs related to cardiovascular and noncardiovascular morbidities. Because there were no differences in the prevalence of noncardiovascular morbidities at the baseline, we assumed that lower cumulative medical costs in the FFR-based PCI group originated from cardiovascular events. However, there remain potential limitations, and the cost-effectiveness of FFR-based PCI should be discussed in a future study.

## Conclusions

Use of FFR in PCI for patients with stable or unstable angina showed significantly lower risk of all-cause death and spontaneous MI, compared to angiography-based PCI. Although initial medical cost was higher in the FFR group, cumulative medical cost after index admission was significantly lower following FFR-based PCI than angiography-based PCI. These results from a nationwide cohort support the current guidelines that FFR should be used in decision-making for PCI and proper lesion selection during PCI (Central Illustration).

Perspectives **COMPETENCY IN MEDICAL KNOWLEDGE:** FFR measurement is recommended to assess hemodynamic significance and guide PCI in patients with IHD. Nevertheless, the penetration rate of FFR in contemporary practice remains limited and physician’s attitude has not been changed probably because of a gap of evidence, especially for 2 remaining questions in daily practice: 1) can FFR-guided PCI reduce patient mortality; and 2) despite initially higher costs after the use of FFR, will cumulative medical costs after index procedure decrease in the long term?

**TRANSLATIONAL OUTLOOK:** In this nationwide cohort study that included 134,613 patients (n = 129,497 in the angiography group and n = 5,116 in the FFR group), FFR-guided PCI showed significantly lower risk of all-cause death or spontaneous MI than the angiography-guided PCI at 4 years. Although the FFR group showed higher medical cost during index admission, cumulative medical cost after index admission was significantly lower in the FFR group than the angiography group based on lower risk of adverse clinical events. These results from a nationwide cohort study support the current guidelines that FFR should be used in decision-making for PCI.

**Central Illustration:**
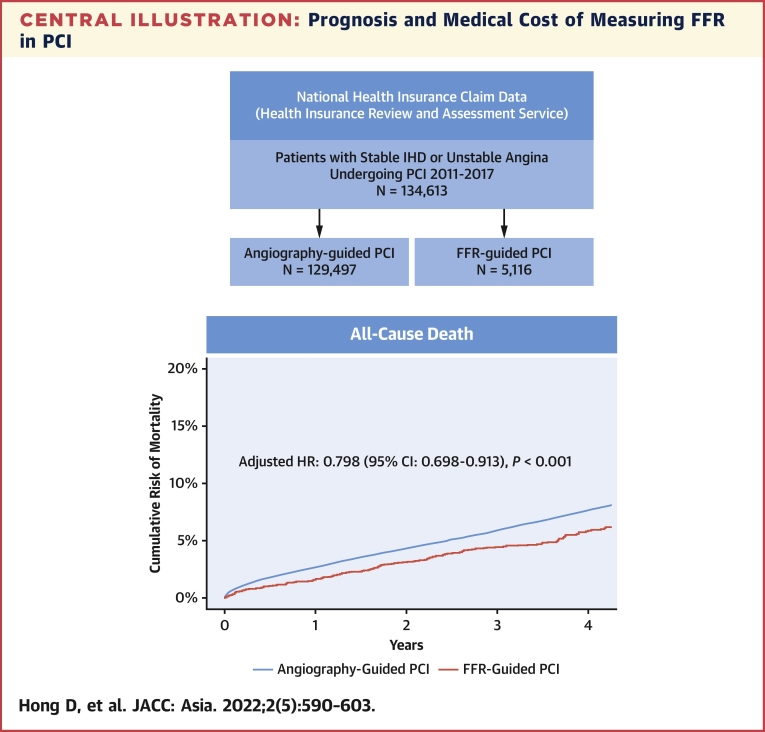

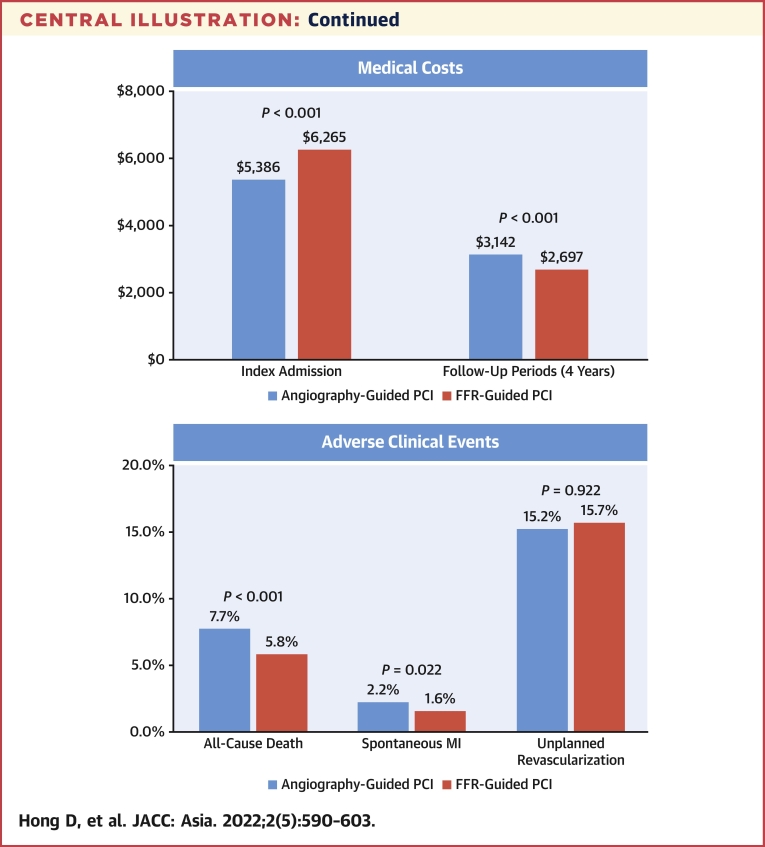
Prognosis and Medical Cost of Measuring FFR in PCI This study evaluated prognosis and medical cost of fractional flow reserve (FFR) use in patients undergoing percutaneous coronary intervention (PCI) for stable ischemic heart disease (IHD) or unstable angina. Among 134,613 patients from the Nationwide Korean National Health Insurance Service database, PCI was guided by angiography (n = 129,497) and FFR (n = 5,116). During the study period, both the number and proportion of FFR-based PCI increased in Korea. The FFR group showed significantly lower risk of all-cause death or spontaneous myocardial infarction than the angiography group at 4 years. Although the FFR group showed higher medical cost during index admission, cumulative medical cost after index admission was significantly lower in the FFR group than angiography group based on lower risk of adverse clinical events. These results from a nationwide cohort support the current guidelines that FFR should be used in decision-making for PCI.

## Funding Support and Author Disclosures

Dr Joo Myung Lee has received research grants from St. Jude Medical (Abbott Vascular) and Philips Volcano. All other authors have reported that they have no relationships relevant to the contents of this paper to disclose.

## References

[1] Neumann F.J., Sousa-Uva M., Ahlsson A. (2019). 2018 ESC/EACTS guidelines on myocardial revascularization. Eur Heart J.

[2] Lawton J.S., Tamis-Holland J.E., Bangalore S. (2022). 2021 ACC/AHA/SCAI guideline for coronary artery revascularization: a report of the American College of Cardiology/American Heart Association joint committee on clinical practice guidelines. J Am Coll Cardiol.

[3] Zimmermann F.M., Ferrara A., Johnson N.P. (2015). Deferral vs performance of percutaneous coronary intervention of functionally non-significant coronary stenosis: 15-year follow-up of the DEFER trial. Eur Heart J.

[4] van Nunen L.X., Zimmermann F.M., Tonino P.A. (2015). Fractional flow reserve versus angiography for guidance of PCI in patients with multivessel coronary artery disease (FAME): 5-year follow-up of a randomised controlled trial. Lancet.

[5] Xaplanteris P., Fournier S., Pijls N.H.J. (2018). Five-year outcomes with PCI guided by fractional flow reserve. N Engl J Med.

[6] Zimmermann F.M., Omerovic E., Fournier S. (2019). Fractional flow reserve-guided percutaneous coronary intervention vs. medical therapy for patients with stable coronary lesions: meta-analysis of individual patient data. Eur Heart J.

[7] Gotberg M., Cook C.M., Sen S., Nijjer S., Escaned J., Davies J.E. (2017). The evolving future of instantaneous wave-free ratio and fractional flow reserve. J Am Coll Cardiol.

[8] Tebaldi M., Biscaglia S., Fineschi M. (2018). Evolving routine standards in invasive hemodynamic assessment of coronary stenosis: the nationwide Italian SICI-GISE cross-sectional ERIS study. J Am Coll Cardiol Intv.

[9] Cheol Seong S., Kim Y.Y., Khang Y.H. (2017). Data resource profile: the National Health Information Database of the National Health Insurance Service in South Korea. Int J Epidemiol.

[10] You S.C., Rho Y., Bikdeli B. (2020). Association of ticagrelor vs clopidogrel with net adverse clinical events in patients with acute coronary syndrome undergoing percutaneous coronary intervention. JAMA.

[11] Shaw L.J., Berman D.S., Maron D.J. (2008). Optimal medical therapy with or without percutaneous coronary intervention to reduce ischemic burden: results from the Clinical Outcomes Utilizing Revascularization and Aggressive Drug Evaluation (COURAGE) trial nuclear substudy. Circulation.

[12] Pijls N.H., De Bruyne B., Peels K. (1996). Measurement of fractional flow reserve to assess the functional severity of coronary-artery stenoses. N Engl J Med.

[13] Parikh R.V., Liu G., Plomondon M.E. (2020). Utilization and outcomes of measuring fractional flow reserve in patients with stable ischemic heart disease. J Am Coll Cardiol.

[14] Volz S., Dworeck C., Redfors B. (2020). Survival of patients with angina pectoris undergoing percutaneous coronary intervention with intracoronary pressure wire guidance. J Am Coll Cardiol.

[15] Windecker S., Stortecky S., Stefanini G.G. (2014). Revascularisation versus medical treatment in patients with stable coronary artery disease: network meta-analysis. BMJ.

[16] Fearon W.F., Nishi T., De Bruyne B. (2018). Clinical outcomes and cost-effectiveness of fractional flow reserve-guided percutaneous coronary intervention in patients with stable coronary artery disease: three-year follow-up of the FAME 2 trial (Fractional Flow Reserve Versus Angiography for Multivessel Evaluation). Circulation.

[17] Fihn S.D., Gardin J.M., Abrams J. (2012). 2012 ACCF/AHA/ACP/AATS/PCNA/SCAI/STS guideline for the diagnosis and management of patients with stable ischemic heart disease: a report of the American College of Cardiology Foundation/American Heart Association task force on practice guidelines, and the American College of Physicians, American Association for Thoracic Surgery, Preventive Cardiovascular Nurses Association, Society for Cardiovascular Angiography and Interventions, and Society of Thoracic Surgeons. J Am Coll Cardiol.

[18] Davies J.E., Sen S., Dehbi H.M. (2017). Use of the instantaneous wave-free ratio or fractional flow reserve in PCI. N Engl J Med.

[19] Gotberg M., Christiansen E.H., Gudmundsdottir I.J. (2017). Instantaneous wave-free ratio versus fractional flow reserve to guide PCI. N Engl J Med.

[20] Lee J.M., Lee S.H., Hwang D. (2020). Long-term clinical outcomes of nonhyperemic pressure ratios: resting full-cycle ratio, diastolic pressure ratio, and instantaneous wave-free ratio. J Am Heart Assoc.

[21] Xu B., Tu S., Qiao S. (2017). Diagnostic accuracy of angiography-based quantitative flow ratio measurements for online assessment of coronary stenosis. J Am Coll Cardiol.

[22] Collet C., Onuma Y., Sonck J. (2018). Diagnostic performance of angiography-derived fractional flow reserve: a systematic review and Bayesian meta-analysis. Eur Heart J.

[23] Kim J., Kang D., Park H. (2020). Long-term β-blocker therapy and clinical outcomes after acute myocardial infarction in patients without heart failure: nationwide cohort study. Eur Heart J.

[24] Kim D., Yang P.S., You S.C. (2021). Treatment timing and the effects of rhythm control strategy in patients with atrial fibrillation: nationwide cohort study. BMJ.

