# Supplementary material for: Prognosis and Medical Cost of Measuring Fractional Flow Reserve in Percutaneous Coronary Intervention
Source: JACC Asia. 2022 Jul 19;2(5):590–603. doi: 10.1016/j.jacasi.2022.04.006 (PMC9743455; doi:10.1016/j.jacasi.2022.04.006)
Supplement: Supplemental Tables 1 and 2 [file mmc1.docx]

**Supplemental Table 1. Codes Used to Define Study Population, Past Medical Histories, Medications, Procedures, and Devices Used in PCI**

| Diagnosis | ICD-10 Codes |
| --- | --- |
| Ischemic heart disease | I20, I21, I25.6 |
| Myocardial infarction | I21 |
| Stable ischemic heart disease | Codes excluding I20.0 among I20, I25.6 |
| Unstable angina | I20.0 |
| Hypertension | I10-13, I15 |
| Diabetes mellitus | E10-14 |
| Hyperlipidemia | E78 |
| Atrial fibrillation | I48 |
| Congestive heart failure | I11.0, I13.0, I13.2, I25.5, I42.0, I42.5-9, I43, I50, I97.1 |
| Chronic renal failure | I12.0, I13.1, N03.2-7, N05.2-7, N18-19, N25.0, Z49, Z94.0, Z99.2 |
| Chronic obstructive pulmonary disease | J43-46 |
| Previous CVA | G45-46, H34.0, I60-69 |
| Peripheral vascular disease | I70-71, I73.1, I73.8-9, I77.1, I79.0, I79.2, K55.1, K55.8-9, Z95.8-9 |
| **Medications** | **ATC Codes** |
| Antiplatelet |  |
| Aspirin | A01DA05, B01AC06, B01AC30, B01AC56, C07FX02-04, C08CA01, C10BX01-02, C10BX04-06, C10BX08, C10BX12, M01BA03, M03BA53, N02AJ02, N02AJ07, N02AJ18, N02BA01, N02BA51, N02BA71, N02BE51, R05X, R06AA57 |
| Clopidogrel | B01AC04, B01AC30, C08CA01 |
| Ticagrelor | B01AC24 |
| Prasugrel | B01AC22 |
| Anticoagulant (Warfarin or NOAC) | B01AA03, B01AE07, B01AF01-03 |
| ACEI or ARBs | C09AA01-16, C09BA01-09, C09BA12-13, C09BA15, C09BB02-07, C09BB10, C09BB12, C09BX01-05, C09CA01-10, C09DA01-10, C09DB01, C09DB02, C09DB04-09, C09DX01-07, C10BX04, C10BX06-07, C10BX10-18 |
| Beta blocker | C07AA01-03, C07AA05-07, C07AA12, C07AA14-17, C07AA19, C07AA23, C07AA27, C07AB01-14, C07AG01-02, C07BA02, C07BA05-07, C07BA12, C07BA68, C07BB02-04, C07BB06-07, C07BB12, C07BB52, C07BG01, C07CA02, C07CA03, C07CA17, C07CA23, C07CB03, C07CB02-03, C07CB53, C07CG01, C07DA06, C07DB01, C07FB02-03, C07FB07, C07FB12-13, C07FX01-06 |
| **Medications** | **ATC Codes** |
| Calcium channel blocker | C07FB02, C07FB03, C07FB07, C07FB12, C07FB13, C08CA01-16, C08CA51, C08CA55, C08CX01, C08DA01, C08DA02, C08DA51, C08DB01, C08EA01-02, C08EX01-02, C08GA01-02, C09BB02-07, C09BB10, C09BB12, C09DB01-09, C09DX03, C10BX03 |
| Nitrate | C01DA08, C01DA14, C01DA58, C01DX12, C01DX16, C05AE02 |
| Statin | A10BH51-52, C10AA01-08, C10BA01-09, C10BX01-18 |
| Ezetimibe | C10BA02, C10BA05, C10BA06, C10AX09, C10BA10 |
| Fenofibrate | C10AB05, C10AB11, C10BA03, C10BA04, C10BA09 |
| **Procedures and Devices** | **Korea HIRA EDI Codes** |
| Percutaneous coronary intervention | M6551-6654, M6561-6567, M6571, M6572, O1876-1877 |
| Coronary artery bypass graft surgery | O1640-1642, O1647-1649, OA640-642, OA647-649 |
| Pressure wire | J6081 |
| Type of devices |  |
| Drug-eluting stent | J5083, J8083 |
| Drug-coated balloon angioplasty | J4080, J8080 |
| Plain old balloon angioplasty | M6551-6552, O1871-1872 |
| Bare metal stent | J5231, J5603, J8231 |
| Bioresorbable vascular scaffold | J5084 |

Abbreviations: ACEI, angiotensin converting enzyme inhibitor; ARB, angiotensin receptor blocker; ATC, anatomical therapeutic chemical; EDI, electronic data interchange; CVA; cerebrovascular accident; HIRA, Health Insurance Review and Assessment service; ICD-10, International Classification of Diseases-10^th^ revision; NOAC, non-vitamin K antagonist oral anticoagulant.

**Supplemental Table 2. Patient and Procedural Characteristics in Propensity Score Matched Group**

|  | **Total**  **(N=20,464)** | **Angiography-based PCI**  **(N= 15,348)** | **FFR-based PCI**  **(N=5,116)** | **SMD before matching** | **SMD after matching** |
| --- | --- | --- | --- | --- | --- |
| **Demographics** |  |  |  |  |  |
| Age, mean (SD), years | 65.5 (10.3) | 65.4 (10.4) | 65.7 (10.0) | 0.081 | 0.025 |
| Female | 6155 (30.1%) | 4,596 (29.9%) | 1,559 (30.5%) | 0.115 | 0.011 |
| **Clinical diagnosis** |  |  |  | 0.244 | 0.035 |
| Stable ischemic heart disease | 13,175 (64.4%) | 9,946 (64.8%) | 3,229 (63.1%) |  |  |
| Unstable angina | 7,289 (35.6%) | 5,402 (35.2%) | 1,887 (36.9%) |  |  |
| **Cardiovascular risk factors** |  |  |  |  |  |
| Hypertension | 14,993 (73.3%) | 11,248 (73.3%) | 3,745 (73.2%) | 0.036 | 0.002 |
| Diabetes mellitus | 10,506 (51.3%) | 7,863 (51.2%) | 2,643 (51.7%) | 0.050 | 0.009 |
| Hyperlipidemia | 15,449 (75.5%) | 11,605 (75.6%) | 3,844 (75.1%) | 0.129 | 0.011 |
| Atrial fibrillation | 1,187 (5.8%) | 872 (5.7%) | 315 (6.2%) | 0.031 | 0.020 |
| Congestive heart failure | 4,574 (22.4%) | 3,425 (22.3%) | 1,149 (22.5%) | 0.010 | 0.003 |
| Chronic renal failure | 1,642 (8.0%) | 1,226 (8.0%) | 416 (8.1%) | 0.035 | 0.005 |
| Chronic obstructive pulmonary disease | 3,270 (16.0%) | 2,447 (15.9%) | 823 (16.1%) | 0.006 | 0.004 |
| Previous CVA | 5,574 (27.2%) | 4,180 (27.2%) | 1,394 (27.2%) | 0.014 | <0.001 |
| Peripheral vascular disease | 4,007 (19.6%) | 2,990 (19.5%) | 1,017 (19.9%) | 0.002 | 0.010 |
| **Baseline medications** |  |  |  |  |  |
| Aspirin | 12,798 (62.5%) | 9,637 (62.8%) | 3,161 (61.8%) | 0.114 | 0.021 |
| P2Y_12_ inhibitor | 8,779 (42.9%) | 6,598 (43.0%) | 2,181 (42.6%) | 0.022 | 0.007 |
| Anticoagulant (warfarin or NOAC) | 532 (2.6%) | 393 (2.6%) | 139 (2.7%) | 0.024 | 0.010 |
| ACEI or ARBs | 8,244 (40.3%) | 6,190 (40.3%) | 2,054 (40.1%) | 0.012 | 0.004 |
| Beta blocker | 8,092 (39.5%) | 6,107 (39.8%) | 1,985 (38.8%) | 0.070 | 0.020 |
| Calcium channel blocker | 8,414 (41.1%) | 6,315 (41.1%) | 2,099 (41.0%) | 0.047 | 0.002 |
| Nitrate | 5,601 (27.4%) | 4,209 (27.4%) | 1,392 (27.2%) | 0.040 | 0.005 |
| Statin | 12,275 (60.0%) | 9,233 (60.2%) | 3,042 (59.5%) | 0.144 | 0.014 |
| Ezetimibe | 2,210 (10.8%) | 717 (4.7%) | 228 (4.5%) | 0.130 | 0.026 |
| Fenofibrate | 945 (4.6%) | 717 (4.7%) | 228 (4.5%) | 0.029 | 0.010 |
| **Medications after index procedure** |  |  |  |  |  |
| Aspirin | 18,891 (92.3%) | 14,085 (91.8%) | 4,806 (93.9%) | 0.100 | 0.084 |
| P2Y_12_ inhibitor | 19,124 (93.5%) | 14,253 (92.9%) | 4,871 (95.2%) | 0.117 | 0.099 |
| Clopidogrel | 18,472 (90.3%) | 13,770 (89.7) | 4,702 (91.9%) | 0.095 | 0.076 |
| Ticagrelor or Prasugrel | 2,273 (11.1%) | 1,674 (10.9%) | 599 (11.7%) | 0.017 | 0.025 |
| Anticoagulant (warfarin or NOAC) | 1,145 (5.6%) | 860 (5.6%) | 285 (5.6%) | 0.028 | 0.001 |
| ACEI or ARBs | 12,832 (62.7%) | 9,705 (63.2%) | 3,127 (61.1%) | 0.079 | 0.044 |
| Beta blocker | 12,393 (60.6%) | 9,400 (61.2%) | 2,993 (58.5%) | 0.048 | 0.056 |
| Calcium channel blocker | 10,586 (51.7%) | 7,849 (51.1%) | 2,737 (53.5%) | 0.035 | 0.047 |
| Nitrate | 8,939 (43.7%) | 6,852 (44.6%) | 2,087 (40.8%) | 0.097 | 0.078 |
| Statin | 18,750 (91.6%) | 13,956 (90.9%) | 4,794 (93.7%) | 0.118 | 0.104 |
| Ezetimibe | 4,783 (23.4%) | 3,454 (22.5%) | 1,329 (26.0%) | 0.150 | 0.081 |
| Fenofibrate | 816 (4.0%) | 647 (4.2%) | 169 (3.3%) | 0.036 | 0.048 |
| **Procedure characteristics** |  |  |  |  |  |
| Number of stents used, mean (SD) | 1.49 (0.79) | 1.49 (0.79) | 1.50 (0.79) | 0.046 | 0.007 |
| Type of device used |  |  |  | 0.159 | 0.023 |
| Drug-eluting stent | 18,947 (92.6%) | 14,219 (92.6%) | 4,728 (92.4%) |  |  |
| Drug-coated balloon angioplasty | 386 (1.9%) | 282 (1.8%) | 104 (2.0%) |  |  |
| Plain old balloon angioplasty | 629 (3.1%) | 464 (3.0%) | 165 (3.2%) |  |  |
| Bare metal stent | 100 (0.5%) | 78 (0.5%) | 22 (0.4%) |  |  |
| Bioresorbable vascular scaffold | 402 (2.0%) | 305 (2.0%) | 97 (1.9%) |  |  |
| **Medical cost, $**^*^ |  |  |  |  |  |
| Index admission, median (IQR), $ | 5,873.0  (4,794.8-7,724.9) | 5,600.0  (4,656.0-7,599.4) | 6,265.1  (5,440.1-7,967.6) | 0.270 | 0.093 |
| During follow-up period, median (IQR), $ | 2,869.3  (1,096.7-7,574.3) | 2,956.9  (1,114.4-7,837.5) | 2,696.5  (1,049.8-6,963.3) | 0.058 | 0.055 |

Values are mean (SD), median (IQR), or n (%).

^*^ Medical cost in Korean Won was converted to US Dollar (1126.56 Korean Won, ₩ = 1 dollar, $).

Abbreviations: ACEI, angiotensin converting enzyme inhibitor; ARB, angiotensin receptor blocker; CVA, cerebrovascular accident; FFR; fractional flow reserve; IQR, interquartile range; NOAC, non-vitamin K antagonist oral anticoagulant; PCI, percutaneous coronary intervention; SD, standard deviation; SMD, standard mean difference.
